# CHOPCHOP: a CRISPR/Cas9 and TALEN web tool for genome editing

**DOI:** 10.1093/nar/gku410

**Published:** 2014-05-26

**Authors:** Tessa G. Montague, José M. Cruz, James A. Gagnon, George M. Church, Eivind Valen

**Affiliations:** 1 Department of Molecular and Cellular Biology, Harvard University, Cambridge, MA 02138, USA; 2 Harvard School of Engineering and Applied Sciences, Cambridge, MA 02138, USA; 3 Wyss Institute for Biologically Inspired Engineering, Harvard University, Cambridge, MA 02138, USA; 4 Department of Genetics, Harvard Medical School, Boston, MA 02115, USA

## Abstract

Major advances in genome editing have recently been made possible with the development of the TALEN and CRISPR/Cas9 methods. The speed and ease of implementing these technologies has led to an explosion of mutant and transgenic organisms. A rate-limiting step in efficiently applying TALEN and CRISPR/Cas9 methods is the selection and design of targeting constructs. We have developed an online tool, CHOPCHOP (https://chopchop.rc.fas.harvard.edu), to expedite the design process. CHOPCHOP accepts a wide range of inputs (gene identifiers, genomic regions or pasted sequences) and provides an array of advanced options for target selection. It uses efficient sequence alignment algorithms to minimize search times, and rigorously predicts off-target binding of single-guide RNAs (sgRNAs) and TALENs. Each query produces an interactive visualization of the gene with candidate target sites displayed at their genomic positions and color-coded according to quality scores. In addition, for each possible target site, restriction sites and primer candidates are visualized, facilitating a streamlined pipeline of mutant generation and validation. The ease-of-use and speed of CHOPCHOP make it a valuable tool for genome engineering.

## INTRODUCTION

The discovery of numerous bacterial nucleic acid modification systems has led to the recent development of two modular, precise genome editing tools (1,2). The TALE (transcription activator-like effector) and CRISPR/Cas (clustered regularly interspaced short palindromic repeats) systems have recently been optimized for research use to site-specifically introduce mutations and manipulate transcriptional activation and repression in a variety of organisms (3–7). 

TALENs are a genome editing method derived from plant pathogenic bacteria (2). TALE architecture is composed of three parts: an N-terminal domain, TALE repeat domains, and a C-terminal domain. The TALE repeat domains typically consist of 34 amino acid residues, where the 12th and 13th repeat variable di-residues (RVDs) determine DNA nucleotide binding specificity (8,9). Each RVD recognizes a specific nucleotide, leading to a simple code for DNA recognition: NI for adenine, HD for cytosine, NG for thymine and NH or NN for guanine (8–11). Importantly, the RVDs can be assembled sequentially to bind any given target sequence. For genome editing purposes, TALEs are fused to the *FokI* nuclease domain to create TALE nucleases (TALENs). Because *FokI* only cleaves as a dimer, sites must be targeted by a pair of TALENs binding on opposite faces of the DNA strand, spaced ∼14–20 bp apart. The *FokI* nuclease domains dimerize across the spacer sequence and create a double-strand break (DSB). The DSB can be repaired through error-prone non-homologous end-joining (NHEJ), which often results in indels and potentially frameshift mutations. For efficient binding, TALEN target sequences require a thymine at the 5′ end for recognition by the TALE N-terminus (3,8,9).

The CRISPR/Cas9 system originates from a bacterial immune system that has been adopted for use as a programmable genome editing tool. *Streptococcus pyogenes* Cas9 nuclease is directed to target sites in the genome by a single-guide RNA (sgRNA) (4,5,12). The Cas9/sgRNA complex binds a 20 bp target sequence followed by a 3 bp protospacer adjacent motif (PAM) -NGG (two invariable Gs preceded by a variable base), and it creates a DSB that is repaired in a seemingly identical manner to TALEN-induced DSBs. While the presence of an -NGG PAM motif is one of the few requirements for binding, the methods used to generate sgRNAs for targeting often impose additional restrictions. Depending on the polymerase used for sgRNA synthesis, the 5′ end dinucleotides may be limited to, for example, 5′ GN- for the commonly used U6 promoter (polymerase III), or 5′ GG- for T7 polymerase (4,5,13). In addition, certain criteria such as guanine-cytosine content (GC-content) appear to influence binding efficiency (14,15). These, along with other guidelines to ensure target suitability, have been used to mostly manually design sgRNAs to generate mutations and knockouts in a variety of organisms including bacteria, yeast, zebrafish, *Xenopus*, nematodes, fruit flies, mice and human cells (4,5,13,16–22).

TALEN and sgRNA design requires identification of target sites that fulfill certain sequence requirements while simultaneously avoiding off-targets elsewhere in the genome. Several studies have demonstrated the limited specificity of TALEN- and particularly Cas9-based genome editing strategies, highlighting the importance of determining the uniqueness of each candidate target site (3,6,23–27). Existing tools for identifying TALEN or sgRNA target sites (25,28-34) have limitations, including acceptance of few input formats, slow search times, restriction to either TALEN or CRISPR/Cas9 target design, minimal or no visualization of the target locus and/or limited information about potential off-target sites (Supplementary Table S1).

We have developed CHOPCHOP, a web-based tool that allows users to easily and rapidly select the optimal TALEN or CRISPR/Cas9 target sequences in genes from a variety of organisms. To overcome limitations of previous tools, CHOPCHOP accepts a wide range of inputs, employs rigorous off-target search algorithms to predict the specificity of each target site in the genome (35), and displays all options in an interactive graphical interface. In addition, to expedite the validation process, CHOPCHOP designs target site-specific primers for polymerase chain reaction (PCR) and displays them together with restriction sites in the gene context.

## MATERIALS AND METHODS

### Target sequence

CHOPCHOP accepts three forms of input: gene name, genomic coordinates or DNA sequence. If the user provides a gene name, CHOPCHOP converts it to genomic coordinates in the relevant organism by consulting gene tables from a variety of sources (e.g. University of California Santa Cruz (UCSC) Genome Browser (36)). If the user provides genomic coordinates, for instance to target an intron, these coordinates (or coordinates from the gene table) are parsed by TwoBitToFa (36), which retrieves the DNA sequence corresponding to the genomic region. If the user provides direct DNA sequence, this sequence (or sequence from TwoBitToFa) is scanned for all potential target sites fulfilling the sequence requirements for the current search (as decided by the user).

### Search for off-targets

The candidate target sites are mapped by Bowtie (35) with the appropriate number of mismatches (‘-v’ mode according to the user-specified options) in a sub-region of the target site where appropriate (‘-L’ seed mode). In TALEN mode, two target sites are paired if they are within a specified range determined by the user. Each sgRNA or TALEN pair is then ranked according to: (i) the number of off-targets in the genome (TALEN mode considers individual off-targets and paired off-targets), and (ii) how many mismatches lie within the off-targets. In addition, for CRISPR/Cas9 mode, the results are ranked by: (iii) GC-content, and (iv) the presence of a guanine at position 20 in the sgRNA target site (14,15). Any target sites with the same score are then sorted by their position in the gene (with preference to 5′ positions). The specific metrics employed by CHOPCHOP are listed on the site under ‘Scoring’. These are updated with new findings from the literature. TALEN results are clustered and suppressed to avoid the display of multiple equivalent TALENs on the results page (e.g. differing by only the size of the spacer sequence). The TALEN pair with the highest ranking in each cluster is displayed on the results page.

### Visualization

Interactive visualization is produced by the D3 JavaScript library (37). The targeted gene or locus is displayed in a zoomable interface, with each sgRNA or TALEN pair displayed at its appropriate location. Clicking on any individual sgRNA/TALEN target site results in a detailed view displaying candidate primer pairs flanking the selected target region and restriction sites.

### Primer design

Primer pairs spanning the target site are designed by the batch version of Primer3 (38) using user-specified options. The default parameters are primers of size 18–25 bp (optimum: 22 bp), a product size of 150–290 bp, and a primer *T*_m_ of 57–63°C (optimum: 60°C). The primers are then mapped to the rest of the genome by Bowtie (35) (options ‘-v 0 –best –k 10’), and subsequently ranked according to their specificity.

## RESULTS

### CHOPCHOP web tool

CHOPCHOP is an easy-to-use web tool that maximizes user flexibility while maintaining a simple and interactive interface. CHOPCHOP can be run in either CRISPR/Cas9 mode or TALEN mode. It runs with default parameters, but accepts a range of advanced options for more refined searches. CHOPCHOP employs a powerful system for finding off-target sites, and displays the output in an interactive table and within the gene architecture. CHOPCHOP also carries out automated primer design to aid with downstream genotyping steps.

### Implementation

#### Input

**Figure 1.**
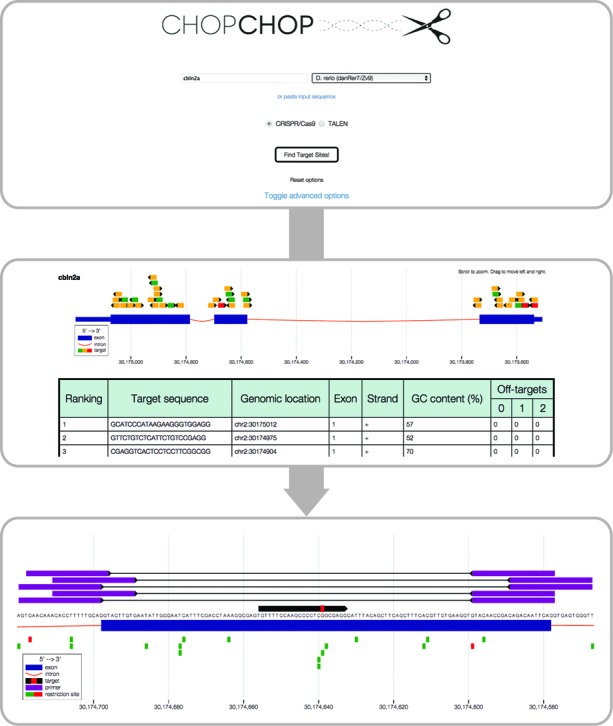
Workflow of a CHOPCHOP CRISPR/Cas9 query. The home page of CHOPCHOP allows users to enter a gene name, genomic coordinates or a DNA sequence, and select an organism and TALEN or CRISPR/Cas9 mode. Advanced options can be toggled. The main results page presents the sgRNA or TALEN target sites within the gene architecture (exon, blue; intron, red), with each option color-coded according to ranking. Hovering over an entry in the table highlights the corresponding graphical sgRNA/TALEN and vice versa. Clicking on a specific result takes the user to a page containing the zoomed in locus with the predicted cut site highlighted in red, primer options in purple and restriction sites color-coded according to whether they are unique in the region.

CHOPCHOP can be run with as few as three basic input options, or with additional advanced parameters. The basic input comprises: (i) a gene name (accepting RefSeq, ENSEMBL, FlyBase and WormBase gene IDs), genomic coordinates or a pasted sequence; (ii) a growing list of organisms (*Homo sapiens*, *Mus musculus*, *Danio rerio*, *Drosophila melanogaster*, *Caenorhabditis elegans*, *Saccharomyces cerevisiae*, *Arabidopsis thaliana*, *Xenopus tropicalis*, *Rattus* *norvegicus, Gallus gallus, Oryzias latipes*, *Gasterosteus aculeatus* and *Anopheles gambiae*); and (iii) the choice between CRISPR/Cas9 or TALEN mode (Figure 1). The advanced options allow the user to target a sub-region of the gene, such as the 5′ UTR, 3′ UTR, splice sites, full exons (including UTRs), or a specified subset of exons.

The CRISPR/Cas9 search mode locates 23 bp target sites including the PAM motif. The user may restrict this search to only target sites suited for synthesis using a particular polymerase, e.g. GG- or GN-/NG- at the 5′ end of the sgRNA. Recent reports have shown that Cas9 can have substantial off-target activity in the genome (23–26) and that tolerance to mismatches shows significant variance depending on the position within the sgRNA (4,25). Another study demonstrated that the number of mismatches tolerated is dependent upon the specific sgRNA (23), suggesting there is no universal rule for CRISPR/Cas9 sgRNA off-target prediction. For this reason, CHOPCHOP offers the choice between a variety of published methods for off-target prediction. (i) One study found mismatches were tolerated at any position except within the PAM motif (25). CHOPCHOP provides a search mode reflecting this rule, searching for mismatches across all bases upstream of the PAM. This is the default mode. (ii) An alternative study found that single-base mismatches up to 11 bp 5′ of the PAM completely abolished cleavage by Cas9 (4). In contrast, mutations further upstream of the PAM retained cleavage activity. CHOPCHOP therefore provides an alternative search mode that locates off-targets with mismatches only in the region where a mismatch would still induce cleavage. (iii) Finally, CHOPCHOP provides a fast mode that only searches for perfect matches of the sgRNA target sequence across the genome.

The TALEN search mode locates pairs of target sites on opposite strands, separated by a 14–20 bp spacer sequence, with the requirement that both sites have a T at the 5′ end. The TALEN-specific options allow the user to cater the target search to a particular TALEN architecture by changing the length of the spacer sequence and the length of the target sites. In addition, depending upon the assembly kit being used, the user can choose to use either the RVD ‘NN’ for guanines, or ‘NH’, which has been shown to bind guanines more specifically than ‘NN’ (10,11). TALEN off-target binding does not appear to have the same position-specific complexity as CRISPR/Cas9 sgRNAs, therefore the TALEN off-target method searches for off-targets with 0, 1 or 2 mismatches across each site. The default method searches for two mismatches.

In order to analyze whether Cas9 or TALENs have successfully cleaved the target locus, users may need to amplify the region of interest for further analysis by methods such as deep sequencing or a T7E1 assay (39). CHOPCHOP therefore integrates primer design with sgRNA/TALEN target site design using Primer3 (38). Primers are designed to amplify the region surrounding the cut site, and mapped against the genome to avoid off-targets producing amplicons of similar length. In the advanced options, the user can adjust the primer specifications, including amplicon size, primer *T*_m_, primer length and the minimum distance between each primer and the target site. In addition, some users might prefer to assess successful mutagenesis using restriction enzyme digestion. CHOPCHOP allows the user to select restriction sites from a particular restriction enzyme company, and it allows the user to specify the minimum size of the restriction site.

#### Output

The majority of CHOPCHOP queries are executed within a matter of seconds, and the results displayed in an interactive table and interactive gene model. CHOPCHOP ranks the search results according to a number of criteria. Both the TALEN and CRISPR/Cas9 modes are ranked by: (i) the number of off-targets, (ii) whether the off-targets contain mismatches or are perfect hits, and (iii) where the target site lies within the architecture of the gene (many users wish to create a frameshift/null mutation and therefore will prefer a mutation at the 5′ end of the gene). Additionally, for CRISPR/Cas9 mode the results are ranked by (iv) GC-content. Recent reports suggest that sgRNAs are most effective with a GC-content between 45 and 80%, and (v) a guanine at position 20 in the target site, which is associated with improved activity (14,15). For TALEN mode, off-targets are specifically scored by whether an individual TALEN target site occurs elsewhere in the genome, or whether both members of a pair lie within cutting distance of one another at an off-target location. For both CRISPR/Cas9 and TALEN mode, the results table provides the sequence of the target site, its ranking, genomic location (including exons and orientation) and the number of potential off-targets with 0, 1 or 2 mismatches (Figure 1). The CRISPR/Cas9 mode also provides the GC-content of the sgRNA target site, and the TALEN mode provides restriction sites that lie in the spacer between two TALENs, as well as the RVDs that should be synthesized for the target site. CHOPCHOP also provides an interactive graphical representation of the gene, with each sgRNA or TALEN target site color-coded according to ranking (Figure 1). This allows users to inspect candidate targets by their location within the gene as well as their specificity within the genome as a whole. The graphical output is generated using the D3 JavaScript library (37) and enables the user to zoom and scroll across the gene. Finally, users can download a text file containing the search results, as well as a GenBank file of the DNA sequence annotated with the target sites, either with or without introns.

**Figure 2.**
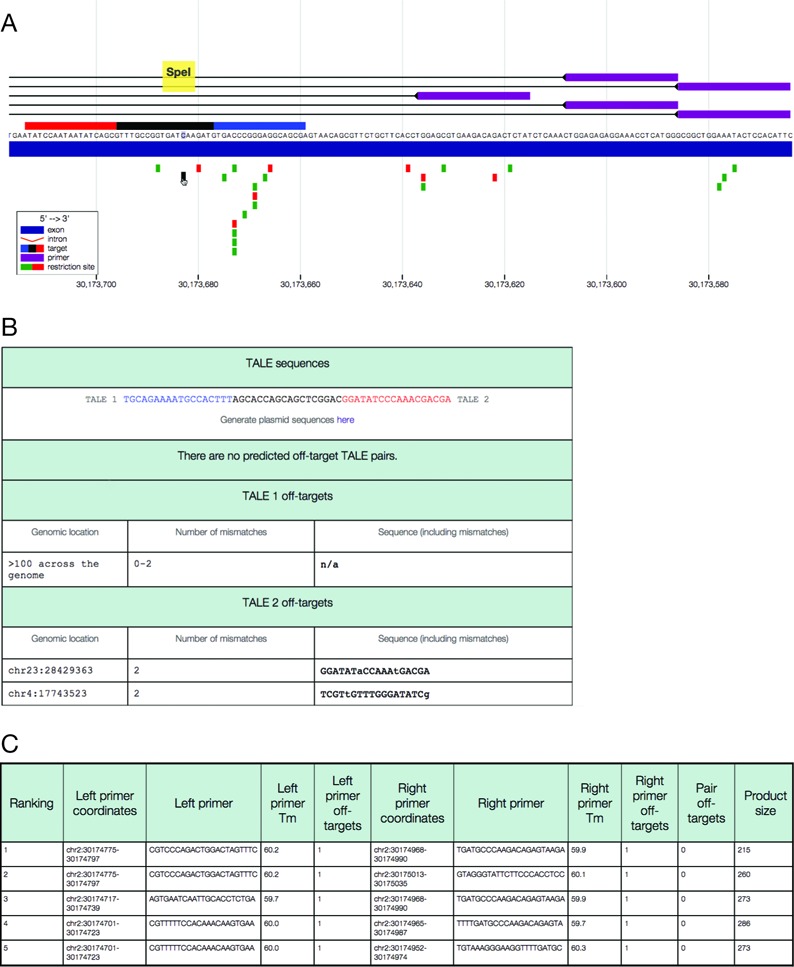
CHOPCHOP provides detailed information about each CRISPR/Cas9 and TALEN target site. (**A**) The detailed information page provides a zoomed in view of the target locus with visible DNA sequence, primer options (above the gene, purple) and restriction sites (below the gene; green if unique in the region, red if not). In TALEN mode the target site is color-coded; TALEN 1 is blue, the spacer is black and TALEN 2 is red. (**B**) Information is provided about predicted off-targets: the genomic location, number of mismatches and location of mismatches within the sequence. (**C**) Information is provided about the primer designs, including the primer sequence, *T*_m_ and product size.

Individual target sites can be inspected in a separate detailed view, displaying additional information about the genomic location of off-targets, and the location of the mismatches within the off-target sites (Figure 2). Upon zooming within this specific region, the DNA sequence becomes visible. For TALENs, the gene view suppresses the visibility of substantially overlapping TALEN pairs to avoid redundancy. In the detailed view, however, all of the clustered targets are listed, should the user prefer a different target sequence in the same approximate location. The detailed view also presents the user with all the restriction sites in the surrounding region that can be used for testing cleavage activity. Restriction sites are color-coded according to whether they are unique within the region. Finally, the detailed view displays primer pairs that flank the target site, and a downloadable GenBank file of the targeted region is available, containing annotations of the target site and primer designs.

## CONCLUSION

CHOPCHOP is a user-friendly web tool that locates optimal CRISPR/Cas9 and TALEN target sites for any genomic region, and presents the information in an interactive and intuitive manner. CHOPCHOP expedites the design process for CRISPR/Cas9- or TALEN-based mutations with fast run times, powerful off-target prediction and integrated primer design.

CHOPCHOP has a number of features that separate it from the other CRISPR/Cas9 or TALEN tools currently available (Supplementary Table S1) (25,28-34). First, CHOPCHOP accepts a wide range of inputs - gene identifiers, genomic regions or pasted sequences - making it suitable for a broad range of uses. Second, CHOPCHOP provides a dynamic graphical output display that includes an interactive visualization of the gene, with each Cas9/TALEN target site displayed at its genomic position and color-coded according to its quality. The visualization of all possible target sites in the gene model makes the selection of the optimal candidate easy, and is an ideal system for the design of two sgRNAs, as used in the increasingly popular dual nickase approach (40). Third, unlike most tools, CHOPCHOP integrates TALEN and CRISPR/Cas9 target design into a single tool. Fourth, CHOPCHOP provides automatic primer generation and restriction site visualization for genotyping. Finally, CHOPCHOP provides downloadable results, including a GenBank file with annotations of the gene's exons, introns and target sites, and a GenBank file of the specific target region with primer designs. CHOPCHOP creates a streamlined process from start to end of mutant design, and is a valuable new resource for genome editing technologies.

## SUPPLEMENTARY DATA

Supplementary Data are available at NAR Online.

Supplementary Data
