# Supplementary material for: CHOPCHOP: a CRISPR/Cas9 and TALEN web tool for genome editing
Source: Nucleic Acids Res. 2014 May 26;42(Web Server issue):W401–7. doi: 10.1093/nar/gku410 (PMC4086086; doi:10.1093/nar/gku410)
Supplement: Supplementary Data [file supp_42_W1_W401__index.html]

Supplementary Data 

# CHOPCHOP: a CRISPR/Cas9 and TALEN web tool for genome editing

## Supplementary Data

**Files in this Data Supplement:**

- Supplemental Figures
