# Supplementary material for: CHOPCHOP: a CRISPR/Cas9 and TALEN web tool for genome editing
Source: Nucleic Acids Res. 2014 May 26;42(Web Server issue):W401–7. doi: 10.1093/nar/gku410 (PMC4086086; doi:10.1093/nar/gku410)
Supplement: Supplementary Data [file supp_gku410_nar-00284-web-b-2014-File004.doc]

|  | **CHOPCHOP** | **CasOT** (26) | **CRISPR Optimal Target Finder** | **E-CRISP** (27) | **E-TALEN** (28) | **CRISPR Design** (24) | **MojoHand** (29) | **TALE-NT** (30) | **ZiFiT** (31) |
| --- | --- | --- | --- | --- | --- | --- | --- | --- | --- |
| **GENERAL** | | | | | | | | | |
| Web tool | X |  | X | X | X | X | X | X | X |
| Cas9 mode | X | X | X | X |  | X |  |  | X |
| TALEN mode | X |  |  |  | X |  | X | X | X |
| Primer design | X |  |  |  |  |  |  |  |  |
| Run time* | <5s | N/A | <10s | <5s | <5s | <15 min | <30s | <2 min | <5s |
| Predicts off-targets | X | X | X | X | X | X |  | X | Cas9 mode only |
| **INPUT** | | | | | | | | | |
| Accepts gene names, inc. RefSeq, ENSEMBL, gene IDs | X |  |  | X | X |  | Only NCBI gene IDs |  |  |
| Accepts genomic coordinates | X |  |  |  |  |  |  |  |  |
| Accepts direct sequence | X | X | X | X | X | Very small accepted input (<250 bp) | X | X | X |
| Targets large range of organisms | X | User must upload the genome | Only arthropods and *C. elegans* | X | X | X | N/A | X | X |
| Target options for sub-regions of the gene | X |  |  | X | X |  |  |  |  |
| Searches secondary off-targets (e.g. GFP) |  |  |  | X | X |  |  |  |  |
| Options for 5’ sequence of sgRNA | X | X | X | X |  |  |  |  | X |
| Options for various Cas9 off-target algorithms, based on current literature | X |  |  |  |  |  |  |  |  |
| TALEN architecture options | X |  |  |  | X |  | X | X | X |
| **OUTPUT** | | | | | | | | | |
| Visualization | X |  | X | X | X | X |  |  |  |
| Ranked list of targets | X | X | X | X | X | X |  | X |  |
| Cas9 mode ranking considers sequence composition of sgRNA target site | X |  |  |  |  |  |  |  |  |
| Downloadable results file | X | X | X | X | X | X | X | X | X |
| Downloadable annotated gene file | X |  |  |  |  | X |  |  |  |
| Visualization of restriction sites | X |  |  |  |  |  |  |  |  |
| Detailed information about off-targets | X | X | X |  |  | X |  |  | X |
| Clusters similar designs to avoid redundancy | X |  |  | X | X |  |  | X | In one mode |

**Supplementary Table S1.** Comparison of features across existing Cas9 and TALEN tools.

*Run time was calculated for a 250 bp *Drosophila* sequence and determined using the network panel of Google Chrome developer tools on a computer running Mac OS X version 10.8.5 with a 1.8 GHz Intel Core i5 processor and 8 GB RAM.
